# MEK inhibitors cobimetinib and trametinib, regressed a gemcitabine-resistant pancreatic-cancer patient-derived orthotopic xenograft (PDOX)

**DOI:** 10.18632/oncotarget.17667

**Published:** 2017-05-07

**Authors:** Kei Kawaguchi, Kentaro Igarashi, Takashi Murakami, Tasuku Kiyuna, Thinzar M. Lwin, Ho Kyoung Hwang, Jonathan C. Delong, Bryan M. Clary, Michael Bouvet, Michiaki Unno, Robert M. Hoffman

**Affiliations:** ^1^ AntiCancer, Inc., San Diego, CA, USA; ^2^ Department of Surgery, University of California, San Diego, CA, USA; ^3^ Department of Surgery, Graduate School of Medicine, Tohoku University, Sendai, Japan

**Keywords:** pancreatic cancer, PDOX, nude mice, orthotopic, drug-response

## Abstract

A pancreatic ductal adenocarcinoma (PDAC), obtained from a patient, was grown orthotopically in the pancreatic tail of nude mice to establish a patient-derived orthotopic (PDOX) model. Seven weeks after implantation, PDOX nude mice were divided into the following groups: untreated control (*n* = 7); gemcitabine (100 mg/kg, i.p., once a week for 2 weeks, *n* = 7); cobimetinib (5 mg/kg, p.o., 14 consecutive days, *n* = 7); trametinib (0.3 mg/kg, p.o., 14 consecutive days, *n* = 7); trabectedin (0.15 mg/kg, i.v., once a week for 2 weeks, *n* = 7); temozolomide (25 mg/kg, p.o., 14 consecutive days, *n* = 7); carfilzomib (2 mg/kg, i.v., twice a week for 2 weeks, *n* = 7); bortezomib (1 mg/kg, i.v., twice a week for 2 weeks, *n* = 7); MK-1775 (20 mg/kg, p.o., 14 consecutive days, *n* = 7); BEZ-235 (45 mg/kg, p.o., 14 consecutive days, *n* = 7); vorinostat (50 mg/kg, i.p., 14 consecutive days, *n* = 7). Only the MEK inhibitors, cobimetinib and trametinib, regressed tumor growth, and they were more significantly effective than other therapies (*p* < 0.0001, respectively), thereby demonstrating the precision of the PDOX models of PDAC and its potential for individualizing pancreatic-cancer therapy.

## INTRODUCTION

Gemcitabine (GEM) is first-line therapy for pancreatic cancer [1, 2] with a poor response rate of approximately 10% [3]. Novel drugs tested on pancreatic cancer include MEK inhibitors (cobimetinib [COB], trametinib [TRA]) [5–8], an PI3K/mTOR inhibitor (BEZ-235) [4–9], an HDAC inhibitor (vorinostat) [10], proteasome inhibitors (bortezomib, carfilzomib) [11, 12], a Wee-1 inhibitor (MK-1775) [13], temozolomide (TEM) [14] and trabectedin (TRAB) [15–17]. Whether a patient's tumor is sensitive to any of these drugs is not knowable *a priory*. Genetic profiling can provide important information, but does not necessarily match drug sensitivity [18].

Clinically-relevant mouse models of pancreatic cancer could enable precision therapy based on the individual patient tumor. For this purpose, our laboratory pioneered the patient-derived orthotopic xenograft (PDOX) nude-mouse model with the technique of surgical orthotopic implantation (SOI), including breast cancer [19], ovarian cancer [20], lung cancer [21], cervical cancer [22], colon cancer [23–25], stomach cancer [26], melanoma [18, 27–29], sarcoma [30–34], as well as pancreatic cancer [35–38]. The PDOX model, developed by our laboratory over the past 28 years, has many advantages, including a patient-like metastatic pattern, over subcutaneous-transplant models which are growing ectopically under the skin and very rarely metastasize [39].

In a previous PDOX study of a BRAF-V600E-mutant melanoma, TRA, an MEK inhibitor, was the only agent of the 4 tested that caused tumor regression. Another MEK inhibitor, COB, could slow but not arrest growth or cause regression of the melanoma. The patient in this study had a BRAF-V600E-mutant melanoma and would be considered to be a strong candidate for vemurafenib (VEM) as first-line therapy, since VEM targets this mutation. However, VEM was not effectivein the PDOX model. The PDOX model thus helped identify the very-high efficacy of TRA against the melanoma PDOX and is a promising drug for this patient. These results demonstrated the powerful precision of the PDOX model for cancer therapy, not achievable by genomic analysis alone [18].

Therefore, in the present study, in a PDOX nude-mouse model with pancreatic cancer from a patient we evaluated TRA and COB and 8 other drugs to find the best treatment strategy for this patient and demonstrate the precision of the pancreatic-cancer PDOX model. As with the melanoma PDOX, TRA and COB were most effective in the pancreatic cancer PDOX.

## RESULTS AND DISCUSSION

**Figure 1:**
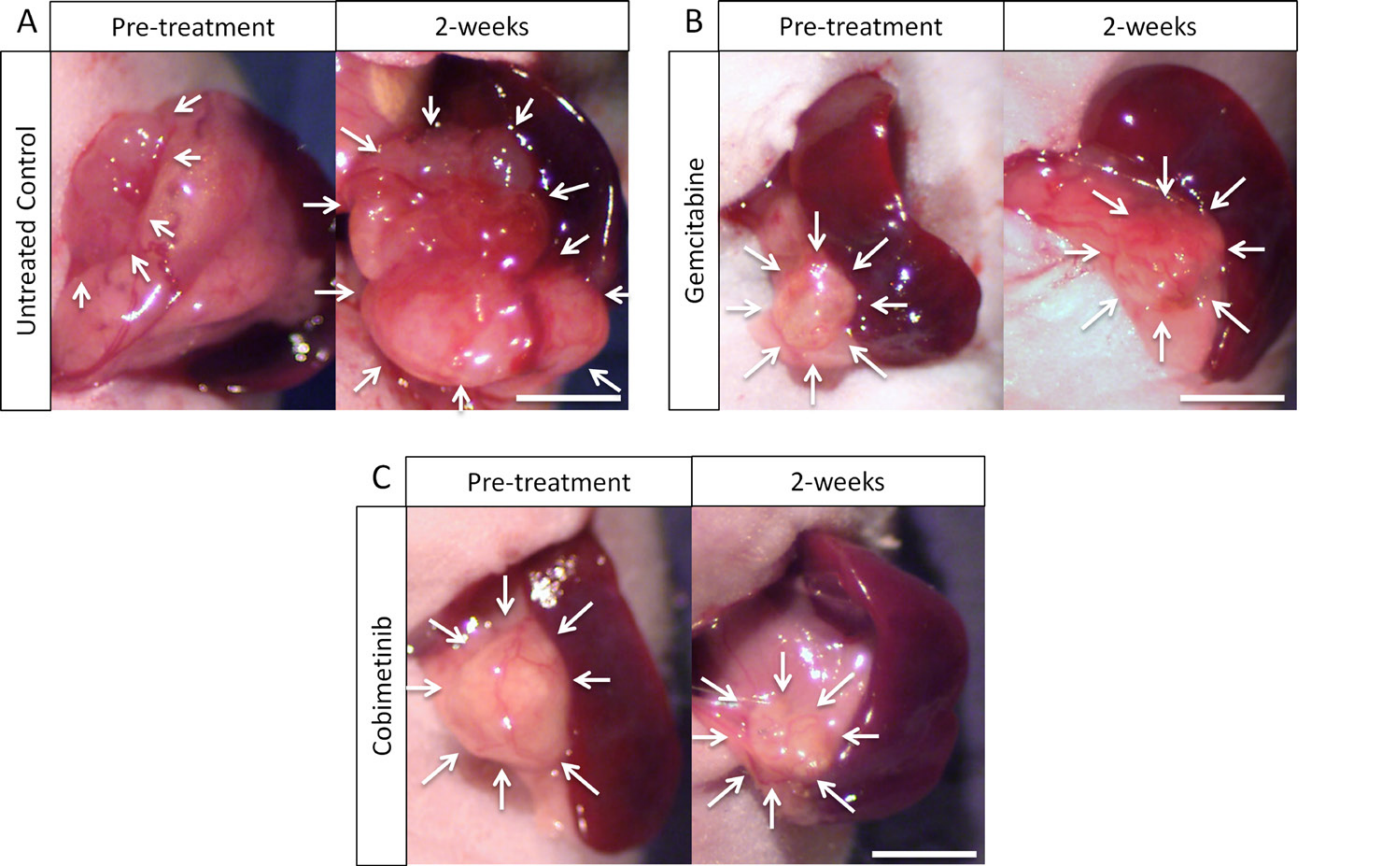
Macroscopic evaluation of therapeutic efficacy (**A**) Control. (**B**) Tumor treated with gemcitabine (GEM). (**C**) Treatment with cobimetinib (COB). White arrows show PDOX tumors on the pancreas. Scale bars: 5 mm.

**Figure 2:**
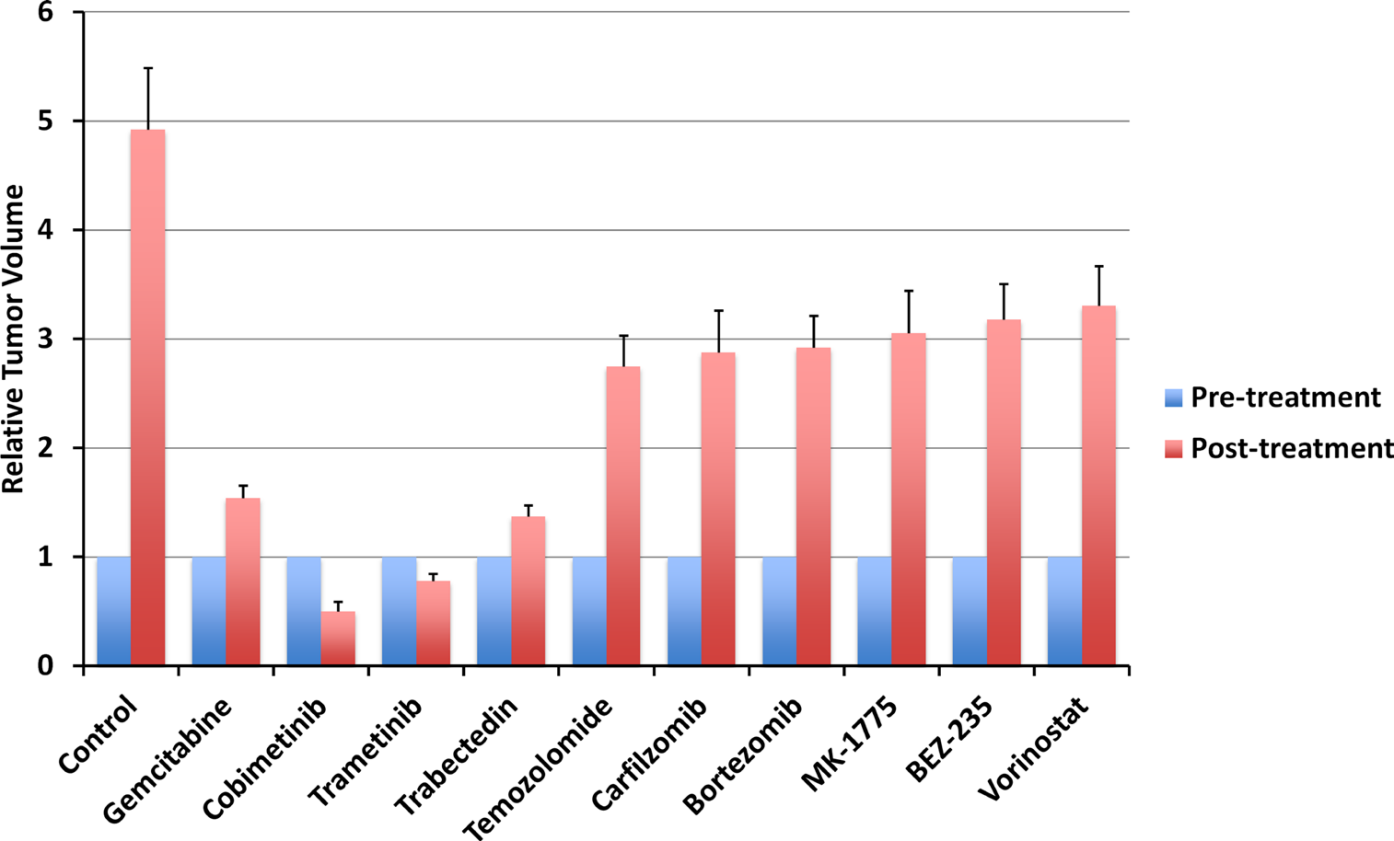
Quantitative treatment efficacy of 10 drugs Line graph shows relative tumor volume at post-treatment relative to pre-treatment tumor volume. All treatments significantly inhibited tumor growth compared to untreated control (*p* < 0.0001). Only MEK inhibitors (COB, TRA) regressed tumor growth. Error bars: ± SD.

All tested drugs, including GEM, COB, TRA, TRAB, TEM, carfilzomib, bortezomib, BEZ-235, vorinostat, inhibited tumor growth in the pancreatic cancer PDOX compared to untreated control (*p* < 0.0001, respectively) on day 14 after treatment initiation. However, only the MEK inhibitors, COB and TRA, regressed tumor growth and they were significantly more effective than other drugs (*p* < 0.0001, respectively) including GEM that is widely used as first line standard therapy for pancreatic cancer (*p* < 0.0001). There was no significant difference between COB and TRA (*p* = 0.0988) (Figures 1, 2).

**Figure 3:**
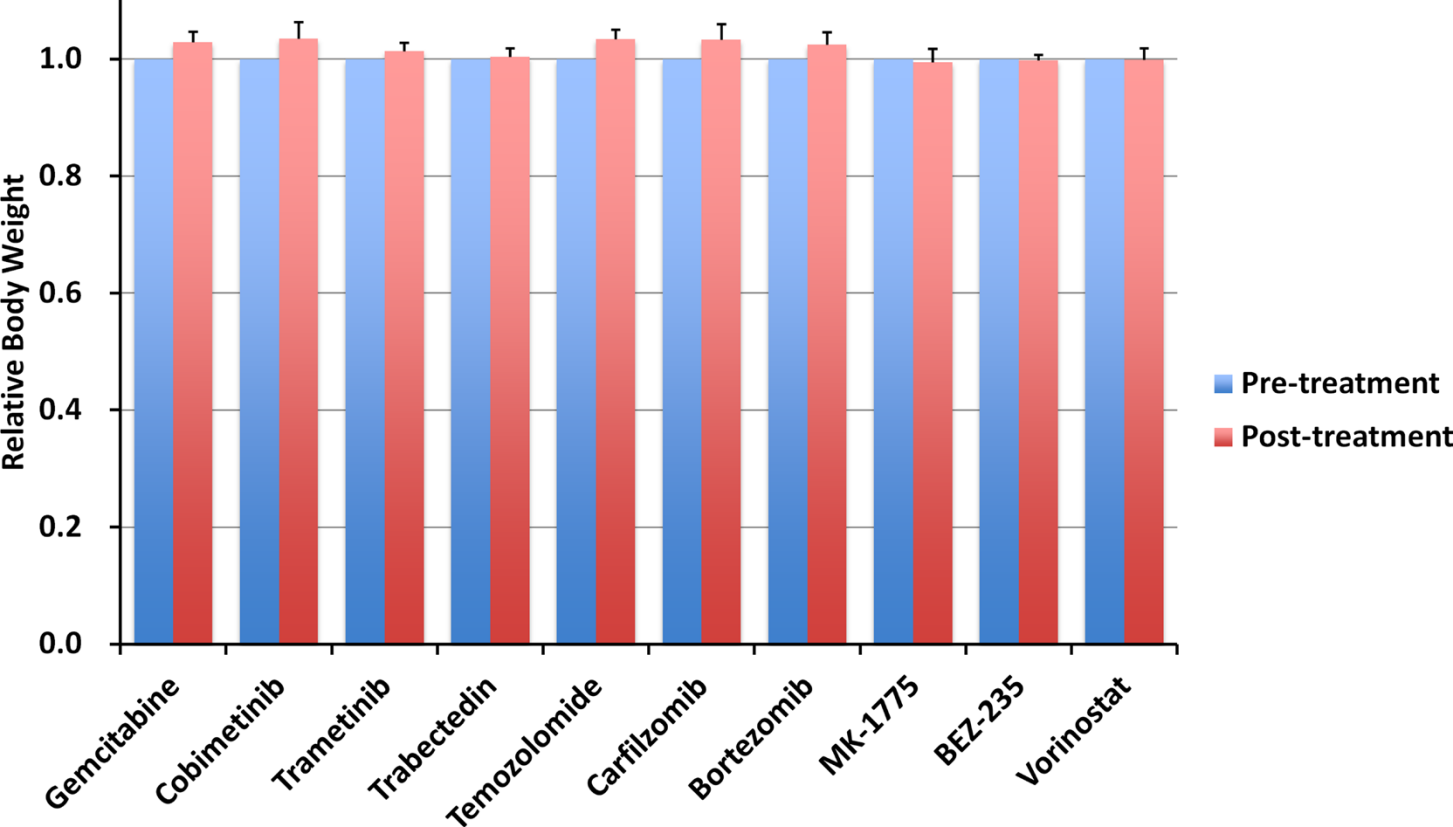
Effect of each drug on mouse body weight Bar graph shows relative body weight in each treatment group at post-treatment relative to pre-treatment. Error bars: ± SD.

The relative body weight on day 14 compared with day 0 did not significantly differ between any treatment group or untreated control (Figure 3). There were no animal deaths in any groups.

**Figure 4:**
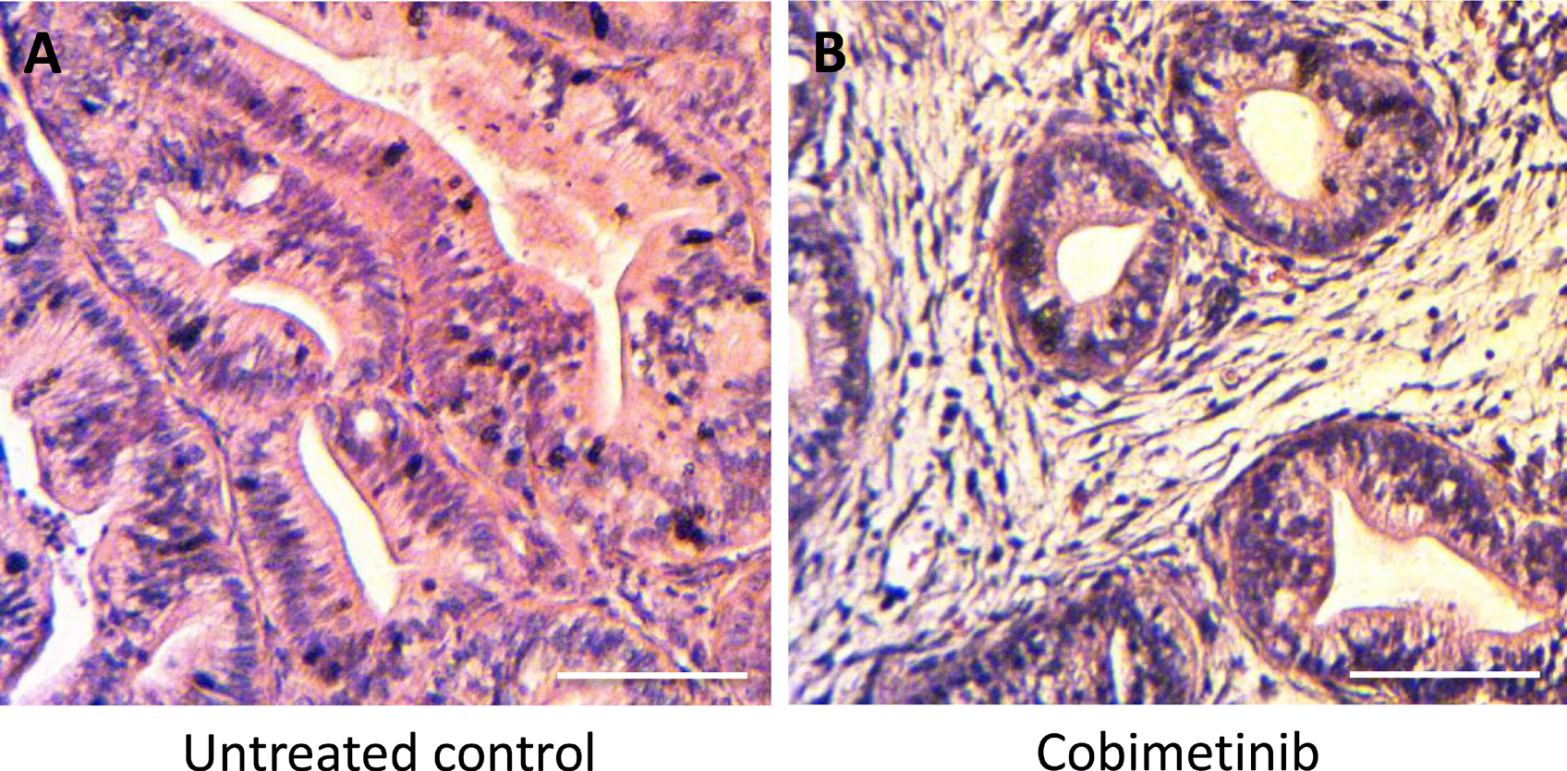
Tumor histology after treatment (**A**) Untreated control. (**B**) Treated with COB. Scale bars: 100 μm

Histologically, the untreated control tumor was mainly comprised of viable cells, in contrast, necrosis was observed in the tumor treated with COB (Figure 4).

GEM is first-line therapy for pancreatic cancer, but the response rate is only approximately 10% [3]. In the present study, GEM could not arrest or regress the tumor growth but showed inhibition compared to the untreated control. In contrast, COB and TRA regressed the tumor and were significantly more effective than GEM. These results suggest that MEK inhibitors might be used as first line therapy for this patient.

Although, the present patient's tumor was sensitive to MEK inhibitors in the PDOX models. Other patients’ tumors may be sensitive for other drugs such as TRAB, TEM, carfilzomib, bortezomib, MK-1775, BEZ-235, or vorinostat, as well as GEM. A PDOX model enables precise, individualized therapy, especially for recalcitrant disease such as pancreatic cancer [18].

Previously-developed concepts and strategies of highly-selective tumor targeting can take advantage of molecular targeting of tumors, including tissue-selective therapy which focuses on unique differences between normal and tumor tissues [40–45].

## MATERIALS AND METHODS

### Mice

Athymic *nu/nu* nude mice (AntiCancer Inc., San Diego, CA), 4–6 weeks old, were used in this study. Animals were housed in a barrier facility on a high efficacy particulate arrestance (HEPA)-filtered rack under standard conditions of 12-hour light/dark cycles. The animals were fed an autoclaved laboratory rodent diet. All mouse surgical procedures and imaging were performed with the animals anesthetized by subcutaneous injection of a ketamine mixture (0.02 ml solution of 20 mg/kg ketamine, 15.2 mg/kg xylazine, and 0.48 mg/kg acepromazine maleate). The response of animals during surgery was monitored to ensure adequate depth of anesthesia. The animals were observed on a daily basis and humanely sacrificed by CO_2_ inhalation if they met the following humane endpoint criteria: severe tumor burden (more than 20 mm in diameter), prostration, significant body weight loss, difficulty breathing, rotational motion, and body temperature drop. All animal studies were conducted in accordance with the principles and procedures outlined in the National Institutes of Health Guide for the Care and Use of Animals under Assurance Number A3873-1 [18, 27–29].

### Patient-derived tumor

The pancreatic cancer was resected in the Department of Surgery, University of California, San Diego (UCSD). Written informed consent was provided by the patient, and the Institutional Review Board (IRB) of UCSD approved this experiment.

### Establishment of PDOX models of pancreatic cancer by surgical orthotopic implantation (SOI)

A fresh sample of pancreatic cancer of the patient was obtained and transported immediately to the laboratory at AntiCancer, Inc., on wet ice. The sample was cut into 5-mm fragments and implanted subcutaneously in nude mice. After five weeks, the subcutaneously-implanted tumors grew to more than 10 mm in diameter. The subcutaneously-grown tumors were then harvested and cut into small fragments (3 mm^3^). After nude mice were anesthetized with the ketamine solution described above, a 1–2 cm skin incision was made on the left side abdomen through the skin, fascia and peritoneum and pancreas was exposed. Surgical sutures (8–0 nylon) were used to implant tumor fragments onto the tail of pancreas to establish the PDOX model [35–38]. The wound was closed with a 6–0 nylon suture (Ethilon, Ethicon, Inc., NJ, USA.

### Treatment study design

PDOX mouse models were randomized into the following groups of 7 mice each: untreated control (*n* = 7); GEM (100 mg/kg, i.p., once a week for 2 weeks, *n* = 7); COB (5 mg/kg, p.o., 14 consecutive days, *n* = 7); TRA (0.3 mg/kg, p.o., 14 consecutive days, *n* = 7); TRAB (0.15 mg/kg, i.v., once a week for 2 weeks, *n* = 7); TEM (25 mg/kg, p.o., 14 consecutive days, *n* = 7); carfilzomib (2 mg/kg, i.v., twice a week for 2 weeks, *n* = 7); bortezomib (1 mg/kg, i.v., twice a week for 2 weeks, *n* = 7); MK-1775 (20 mg/kg, p.o., 14 consecutive days, *n* = 7); BEZ-235 (45 mg/kg, p.o., 14 consecutive days, *n* = 7); vorinostat (50 mg/kg, i.p., 14 consecutive days, *n* = 7). Tumor length and width were measured both pre- and post-treatment. Tumor volume was calculated with the following formula: Tumor volume (mm^3^) = length (mm) × width (mm) × width (mm) × 1/2. Data are presented as mean ± SD. The tumor volume ratio is defined at the tumor volume at a post-treatment point relative to pre-treatment tumor volume.

### Imaging of the pancreatic cancer PDOX model

Imaging of the macroscopic tumor was performed with the OV100 Small Animal Imaging System (Olympus, Tokyo, Japan).

### Histological examination

Fresh tumor samples were fixed in 10% formalin and embedded in paraffin before sectioning and staining. Tissue sections (5 μm) were deparaffinized in xylene and rehydrated in an ethanol series. Hematoxylin and eosin (H&E) staining was performed according to standard protocols. Histological examination was performed with a BHS System Microscope (Olympus Corporation, Tokyo, Japan). Images were acquired with INFINITY ANALYZE software (Lumenera Corporation, Ottawa, Canada) [18, 29].

### Statistical analysis

JMP version 11.0 was used for all statistical analyses. Significant differences for continuous variables were determined using the Mann-Whitney *U* test. Line graphs express mean values and error bars show standard deviation (SD). A probability value of *P* ≤ 0.05 was considered statistically significant.

## CONCLUSIONS

In the present study, the PDOX model identified the ability of MEK inhibitors to regress a pancreatic cancer PDOX. That COB and TRA caused tumor regression indicated their potential efficacy for the patient donor of the PDOX in the present study. These results demonstrate the powerful precision of the PDOX model to distinguish the most effective of the 10 drugs tested.

Future experiments will test the present and other pancreatic cancer PDOX models with various therapies and compare the results with clinical outcome.
